# A case of adult-onset Still disease complicated by macrophage activation syndrome and toxic epidermal necrolysis

**DOI:** 10.1016/j.ero.2025.11.003

**Published:** 2025-12-04

**Authors:** Haojie Xu, Yuan An, Huaqun Zhu

**Affiliations:** Department of Rheumatology and Immunology, Peking University People’s Hospital, Beijing, China

## Abstract

A 56-year-old woman with 1-year recurrent fever, rash, joint swelling (acute exacerbation) was diagnosed with adult-onset Still disease (AOSD) per 1992 Yamaguchi criteria. She initially improved with methylprednisolone, tocilizumab, methotrexate, and iguratimod but relapsed after discontinuing methylprednisolone. Twenty-four hours after first Re Du Ning injection, she developed progressive rash (extensive polymorphic erythema, bullae, and >30% body surface epidermal detachment), high fever, organ impairment, and coagulopathy and was diagnosed with AOSD complicated by macrophage activation syndrome (2004 haemophagocytic lymphohistiocytosis guidelines), toxic epidermal necrolysis, myocardial injury, hepatic impairment, and disseminated intravascular coagulation. Treatment included dexamethasone, methylprednisolone, plasma exchange, cyclosporine, anakinra, ganciclovir, voriconazole, intravenous immunoglobulin, and supportive care, leading to marked improvement in lesions. This case highlights rare AOSD complications and anakinra’s efficacy.

## CASE PRESENTATION

A 56-year-old woman was referred to the rheumatology department for 1-year recurrent fever, rash, joint swelling, and a recent acute exacerbation. Key symptoms included high fever (maximum, 39.0°C), sore throat, rash (Fig, A), polyarthralgia, and lymphadenopathy. Laboratory results revealed a white blood cell count of 18.31 × 10^9^/L (reference, 4-10 × 10^9^/L), absolute neutrophil count of 17.05 × 10^9^/L (reference, 2-7.5 × 10^9^/L), elevated erythrocyte sedimentation rate (ESR) of 75 mm/h (reference, 0-20 mm/h), C-reactive protein (CRP) of 160.9 mg/L (reference, 0-8 mg/L), and ferritin of 38,713 ng/mL (reference, 12-150 ng/mL); antinuclear antibody test returned a titre of 1:80 with a cytoplasmic reticular pattern, and rheumatoid factor was negative. Positron emission tomography/computed tomography showed diffuse uptake in spleen, bone marrow, and lymph nodes, with no infection or malignancy. She was diagnosed with adult-onset Still disease (AOSD) per 1992 Yamaguchi classification criteria [1].

**Figure:**
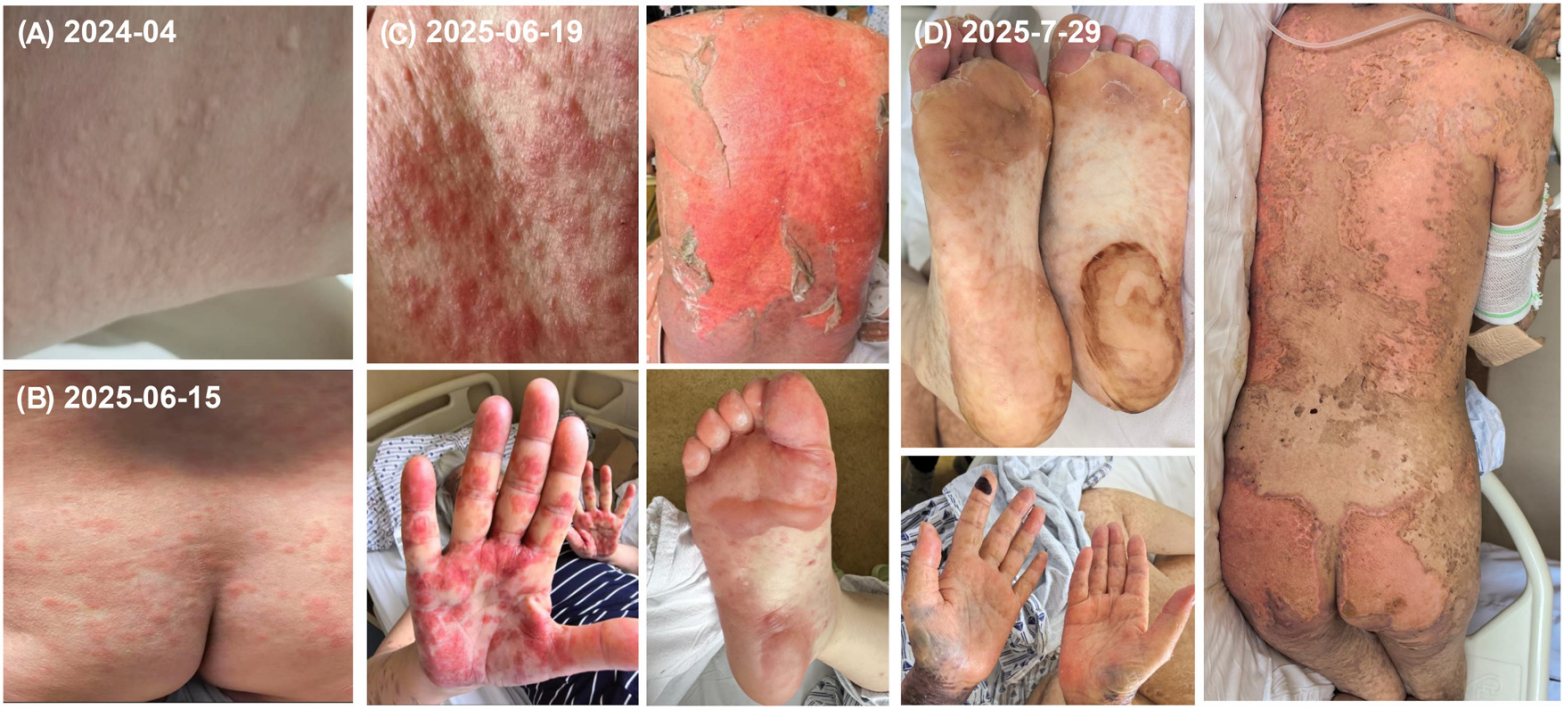
Serial morphological changes of skin lesions during disease progression. (A) Mild urticarial rash. (B) Rash slightly worse, but no severe skin damage. (C) Severe rash with back skin exfoliation and plantar blisters (toxic epidermal necrolysis feature). (D) Rashes improved significantly.

Initial treatment with methylprednisolone, tocilizumab, methotrexate, and iguratimod improved her condition, but her disease relapsed after she self-discontinued methylprednisolone. One week before admission, 24 hours after her first intravenous infusion of Re Du Ning injection (a traditional Chinese medicine containing extracts of *Artemisia annua*, honeysuckle, and gardenia), she developed a progressive rash that advanced into extensive polymorphic erythema with bullae on the back and extremities (Fig, B, C). A detailed medication review confirmed that no drugs known to induce toxic epidermal necrolysis (TEN), including nonsteroidal anti-inflammatory drugs (NSAIDs) and antimicrobial agents, had been administered during this period. Serological tests conducted upon admission (after Re Du Ning administration) yielded negative results for influenza A/B virus nucleic acid, Epstein-Barr virus capsid antigen antibody, and cytomegalovirus (CMV) immunoglobulin (Ig)M; the procalcitonin level was 0.08 ng/mL (reference, <0.5 ng/mL), ruling out active infection as a potential trigger. The patient had no history of using traditional Chinese medicine injections. She had over 30% body surface area (BSA) epidermal detachment (Fig, C); high fever (maximum, 41.0°C); elevated hepatic and myocardial enzymes; prolonged prothrombin time; and increased CRP, ESR, and ferritin levels (Table). Cytokine profiling revealed elevated interleukin (IL)-6 (93.48 pg/mL; reference, 0-11.09 pg/mL), IL-8 (23.57 pg/mL; reference, 0-15.71 pg/mL), interferon gamma (7.6 pg/mL, reference, 0-4.43 pg/mL), and IL-10 (7.8 pg/mL; reference, 0-4.5 pg/mL); other findings included hypertriglyceridemia (2.28 mmol/L; reference, 0.45-1.7 mmol/L), a progressive decline in fibrinogen (≤1.5 g/L) (Table), coagulopathy, reduced natural killer (NK) cell activity (9.68%; reference, 15.11%-23.7%), and elevated soluble CD25 levels (81,030 pg/mL; reference, 0-6400 pg/mL). Serology tests yielded negative results for antineutrophil cytoplasmic antibodies, anti–double-stranded DNA, anticyclic citrullinated peptide, and anti–Jo-1 antibodies. Bone marrow biopsy demonstrated grade IV to V hypocellularity and haemophagocytosis, fulfilling haemophagocytic lymphohistiocytosis (HLH) 2004 guidelines [2]. Final diagnoses were AOSD complicated by macrophage activation syndrome (MAS) and TEN with concomitant myocardial injury, hepatic impairment, and disseminated intravascular coagulation (DIC).

**Table:** Evolution of selected laboratory parameters during pharmacotherapy

ALI, acute liver injury; ALT, alanine aminotransferase; AOSD, adult-onset Still disease; AST, aspartate aminotransferase; BNP, B-type natriuretic peptide; bid, bis in die (twice daily); CRP, C-reactive protein; DIC, disseminated intravascular coagulation; ESR, erythrocyte sedimentation rate; IV, intravenous injection; IVGTT, intravenous glucose tolerance test; MAS, macrophage activation syndrome; MP, methylprednisolone; po, per os (by mouth); qAM, every morning; qPM, every afternoon; qd, quaque die (once daily); q12h, every 12 hours; sc, subcutaneous injection; TEN, toxic epidermal necrolysis; TNI, troponin I; WBC, white blood cell count.

## TREATMENT

On admission, she received intravenous dexamethasone (10 mg every morning and 5 mg every evening for 7 days, followed by a 5-day tapering schedule), methylprednisolone (40 mg/d, intravenous infusion for 8 days, and then switched to oral methylprednisolone at 36 mg/d). For severe DIC (fibrinogen ≤ 1.5 g/L; prolonged prothrombin time) and extensive epidermal detachment (≥30% BSA), 3 plasma exchange sessions were done to eliminate proinflammatory cytokines. She also was treated with cyclosporine (100 mg every 12 hours for 7 days, followed by oral cyclosporine 75 mg twice daily for 14 days). Anakinra was given at a dose of 100 mg daily for 21 days. The reasons for choosing anakinra are as follows: firstly, the 2024 European Alliance of Associations for Rheumatology (EULAR) guidelines recommend IL-1 inhibitors like anakinra for severe AOSD cases with MAS [3]. Secondly, in patients with MAS, tocilizumab (an IL-6 antagonist) is contraindicated, and anakinra has a lower impact on infection [4]. It also has minimal effects on blood cell counts and liver and kidney functions, which is crucial considering the patient’s complex condition. Thirdly, high-dose glucocorticoids/cyclosporine failed to control life-threatening MAS, and anakinra targets IL-1–mediated hyperinflammation (indicated by hyperferritinemia and defective NK cell function) to address refractory inflammation with low infection risk.

CMV DNA (4.52 × 10^3^ copies/mL; reference, ≤10^3^ copies/mL) and galactomannan test (151.6 pg/mL; reference, ≤60 pg/mL) showed positive results; antiviral treatment with ganciclovir and antifungal treatment with voriconazole were initiated for 3 days. Posttreatment, CMV DNA decreased to <10^3^ copies/mL and galactomannan to 45 pg/mL; cyclosporine was reintroduced at a dosage of 75 mg twice daily as infection was controlled. Adjunctive therapies included intravenous Ig (IVIg), hepatoprotective agents, and transfusion support.

Clinical improvement included resolved facial/truncal/extremity cutaneous lesions (Fig, D); normalised inflammatory markers (ESR, CRP, ferritin, and D-dimer); and recovered haematologic, hepatic, and cardiac enzyme parameters. Markers improved were as follows: ferritin, 1516 ng/mL; D-dimer, 466 mg/L; alanine transaminase, 45 U/L (from 130 U/L); and aspartate transaminase, 30 U/L (from 193 U/L) (Table). Polymorphic erythema or bullae crusted on treatment day 5; epidermal detachment resolved completely on day 14 (Fig, D).

## DISCUSSION

AOSD is an autoimmune inflammatory disorder, with MAS occurring in approximately 8% to 15% of cases [5,6]; however, the concurrent TEN and DIC are extremely rare. This case offers valuable insights into the diagnosis and management of life-threatening complications associated with AOSD. Glucocorticoids are considered first-line treatment for most AOSD-related complications, and in this instance, the patient experienced relapse following discontinuation of methylprednisolone. The pathogenesis of AOSD remains incompletely understood, and lymphocytes and monocytes are known to play pivotal roles. Functional defects in NK cells and cytotoxic T lymphocytes may allow for excessive activation of the monocyte-macrophage system upon antigenic stimulation, thereby triggering inflammatory cytokine storms that can culminate in MAS. In this instance, the onset of MAS coincided temporally with TEN, after administration of Re Du Ning injection, further increasing the severity of the condition.

Re Du Ning is a commonly used traditional Chinese medicine injection approved by China’s National Medical Products Administration (approval number: Z20050217) for the treatment of inflammatory conditions. Its main active components consist of artemisinin derivatives (from *A annua*), extracts from honeysuckle (eg, chlorogenic acid), and gardenoside (from gardenia). Pharmacological studies suggest that Re Du Ning demonstrates moderate anti-inflammatory effects similar to NSAIDs by inhibiting cyclooxygenase-2 activity and reducing prostaglandin E2 synthesis [7]. It frequently used as a complementary therapy for infectious fever and inflammatory conditions. In this patient, the temporal association (onset 24 hours postadministration), along with the exclusion of other potential triggers, supports a probable causal relationship between Re Du Ning injection and TEN. A retrospective study of 56 patients with Stevens-Johnson syndrome (SJS) or TEN indicated that antibiotics were the most common sensitising agents (31.51%), followed by NSAIDs (21.92%). Traditional Chinese medicines and health supplements constituted 15.07%, ranking them as the third most frequent category of causative agents [8]. Another study focusing on drug hypersensitivity in autoimmune diseases further highlighted that the incidence of drug allergies in patients with AOSD ranges from 17.6% to 54%, with a heightened susceptibility to developing SJS or TEN [9]. This may be attributed to underlying immune dysregulation in AOSD, such as impaired NK cell function; reduced regulatory T cell numbers; and elevated levels of proinflammatory cytokines like IL-6, IL-1, and IL-18. These factors may predispose patients to heightened T cell activation in response to drug components, such as chlorogenic acid or artemisinin derivatives in Re Du Ning, ultimately triggering TEN.

Clinicians should monitor for cutaneous reactions closely when administering this agent, particularly in patients with underlying autoinflammation diseases who may exhibit heightened immune reactivity. Diagnostic confirmation of MAS was established through characteristic clinical presentation, laboratory parameters, and haemophagocytosis on bone marrow biopsy. Concurrently, the patient developed severe cutaneous drug reaction that progressed to TEN. Notably, MAS and TEN share overlapping pathogenic mechanisms, particularly concerning elevated serum granulysin and impaired T cell autoregulation [10].

Within this context of immune dysregulation, extensive epidermal detachment manifested. Furthermore, concurrent use of glucocorticoids and immunosuppressive therapy significantly increased the risk of infection, necessitating meticulous antimicrobial prophylaxis and management throughout the treatment course. The management of secondary MAS prioritises control of the underlying condition alongside HLH-2004 protocol implementation (typically etoposide, dexamethasone, and cyclosporine) [2]. This patient received aggressive antimicrobial therapy for infection control, glucocorticoids for anti-inflammation purposes, and IVIg along with cyclosporine for immunomodulation. Despite these interventions, myocardial injury, acute hepatic impairment, and DIC ensued. Plasmapheresis provided partial clinical improvement without achieving complete disease control.

Current EULAR/Paediatric Rheumatology European Society guidelines recommend early biologics for AOSD [3]. Given the significant inflammation activation associated with TEN, anakinra was administered. The favourable clinical outcome underscores the therapeutic efficacy of proinflammatory cytokine blockade in disease management. AOSD with MAS/TEN is life-threatening and complicates treatment, and timely multidisciplinary intervention is critical for survival.

This case has several limitations. First, the optimal number of plasma exchange sessions for AOSD-related DIC and TEN remains uncertain, the decision to administer 3 sessions was based on clinical response rather than evidence-based criteria. Second, the duration of anakinra therapy for MAS complicated by TEN is not standardised, necessitating long-term follow-up to evaluate relapse risk following discontinuation. Future studies should establish personalised treatment algorithms for AOSD with multiple life-threatening complications, particularly focusing on the role of IL-1 inhibitors in balancing anti-inflammatory efficacy and infection risk.

## Contributors

HX wrote the original draft. YA reviewed and edited the manuscript. HZ reviewed and edited the manuscript and supervised and conceptualised the study.

## Funding

This study was supported by the National Natural Science Foundation of China (No.82402103), Beijing Natural Science Foundation [No.: 7244425] and Peking University People’s Hospital Scientific Research Development Funds [No.: RDJP2023-01 and RDL2024-17].

## Competing interests

All authors declare they have no competing interests.

## Patient consent for publication

Consent was obtained directly from the patient.

## Ethics approval

Ethical approval was obtained for this case report.

## Provenance and peer review

Not commissioned; externally peer reviewed.

## CRediT authorship contribution statement

**Haojie Xu:** Writing – original draft, Visualization, Conceptualization. **Yuan An:** Writing – review & editing. **Huaqun Zhu:** Writing – review & editing, Conceptualization.
